# Subacute intestinal obstruction by enterolith: a case study

**DOI:** 10.1186/s40064-016-3150-0

**Published:** 2016-08-31

**Authors:** V. S. Kappikeri, Akshay Mahesh Kriplani

**Affiliations:** Department of Surgery, M.R. Medical College, Kalaburagi, 585101 India

**Keywords:** Enterolith, Subacute intestinal obstruction, Ileo-ileal anastomoses

## Abstract

**Introduction:**

Enteroliths are an uncommon entity in humans and form a rare cause of subacute intestinal obstruction. They occur proximal to stricture or in a diverticulum or a blind loop due to stasis.

**Case description:**

40 years male presenting with intermittent right lower abdominal pain since 1 year. Barium meal follow-through and CT abdomen diagnosed as a case of subacute intestinal obstruction with an enterolith in the distal ileal segment. On exploratory laparotomy multiple ileal strictures with mesenteric adhesions were noted. An enterolith was delivered from one of the segments between two strictures followed by segmental resection of the pathological ileal segment with ileo-ileal anastomoses. Histopathology of the ileal segment showed inflammatory infiltrates.

**Discussion and Evaluation:**

It was a case of a primary enterolith formed due to stasis between ileal strictures causing subacute intestinal obstruction.

**Conclusion:**

An enterolith should also be considered while evaluating a case of intestinal obstruction as one of the rare differentials.

## Background

Subacute intestinal obstruction is a common surgical emergency. Common causes are stricture, polyps and tumours, rarely gall stones or foreign body. Enteroliths are also a cause of intestinal obstruction but are rare. They occur proximal to stricture or in a diverticulum or a blind loop. Stasis is an important factor in their production (Yadav et al. 2015). Enteroliths were first described by Pfahler and Stamm in 1915 (D’souza et al. 2010). Here we present a case of subacute intestinal obstruction with multiple strictures and enterolith in the distal ileum, who underwent segmental ileal resection and ileo-ileal anastomoses.

## Case report

**Fig. 1:**
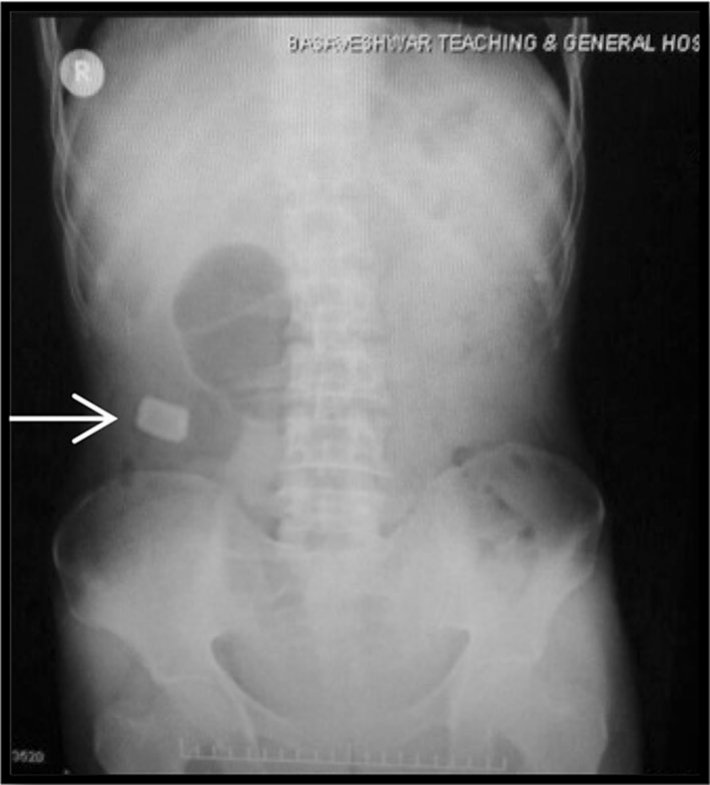
Erect X ray abdomen showing radiopaque shadow in the right iliac fossa

**Fig. 2:**
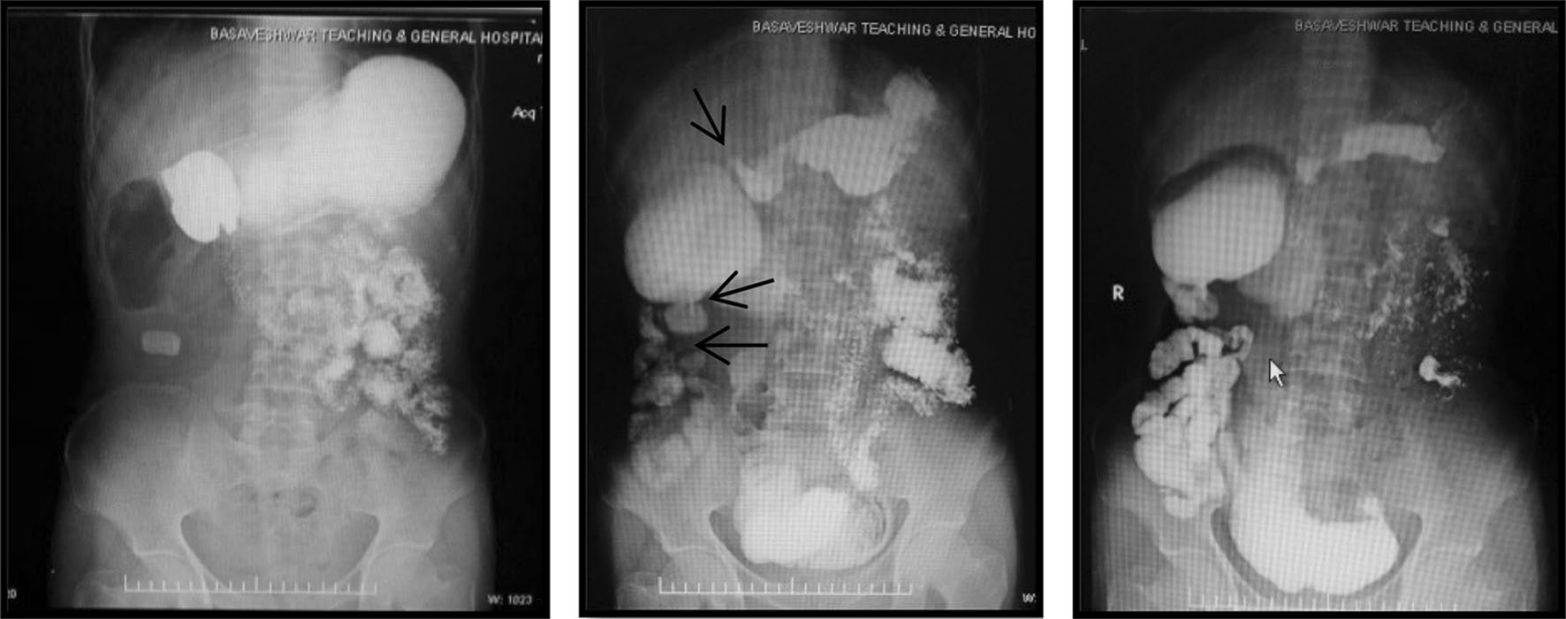
Barium Meal follow through showing radiopaque shadow in right iliac fossa, multiple strictures (*arrows*), dilated proximal intestinal segment with distal collapsed segment

A 40 years male presented with intermittent right lower abdominal pain since 1 year. The pain was dull aching and aggravated with food intake. There was no history of vomiting, fever or chronic cough. Patient had normal bladder and bowel habits. On clinical examination the abdomen was not distended, soft with tenderness in the right iliac fossa. There was no mass palpable. Bowel sounds were well heard. Blood parameters were normal. The ultrasound abdomen showed dilated small bowel loops with a hyperechoic shadow in the small intestine in the right iliac fossa, suggesting an enterolith or a migrated gall stone. An X-ray abdomen in erect posture showed a radiopaque mass in the right iliac fossa with a dilated segment of small bowel, seen as a localised gas shadow on the X-ray (Fig. 1). A contrast X-ray barium meal follow through showed dilated proximal loops of small bowel with multiple strictures in terminal ileum and a rectangular radiopaque shadow in right iliac fossa. Barium transit was slow. Two dilated loops in between strictures were noted. A grossly dilated proximal ileal segment and collapsed distal segment was noted (Fig. 2). A CECT abdomen showed similar findings.

**Fig. 3:**
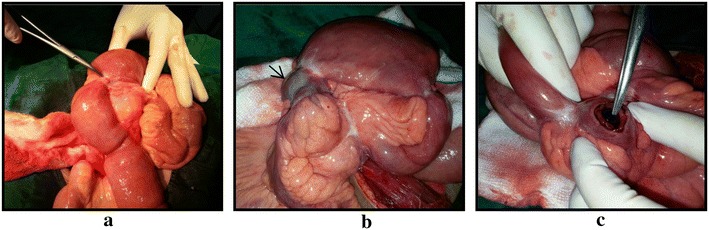
**a** Multiple ileal strictures.** b** Dilated blind loop segments with bowel discolouration of the distal blind loop (*arrow head*).** c** Enterotomy incision with enterolith

**Fig. 4:**
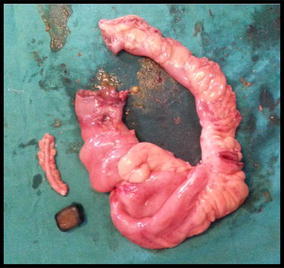
The resected ileo-ileal specimen with appendix and the enterolith

**Fig. 5:**
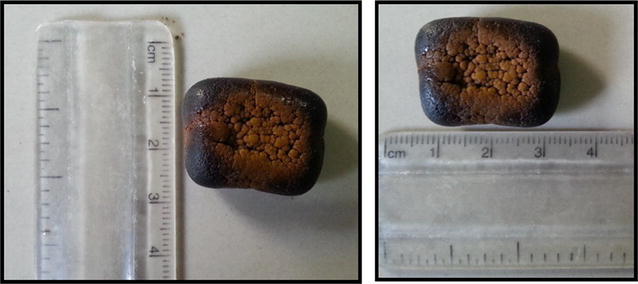
Enterolith measuring 2.5 × 1.5 cm

On exploratory laparotomy multiple strictures were noted in terminal ileum with dilated proximal loops and collapsed distal segment. Mesenteric thickening and adhesions between the small bowel were present (Fig. 3a, b). Two dilated segments between strictures were noted with proximal segment being grossly dilated. The distal segment had bowel wall discolouration. On palpation an enterolith was palpated in the distal segment (Fig. 3b). Enterolith was delivered through an enterotomy incision over the distal segment (Fig. 3c). The pathological ileal segment consisting of multiple strictures was resected and an end to end ileo-ileal anastomoses was done, along with appendicectomy (Fig. 4). There was no fistula seen between gall bladder and duodenum. The rest of the bowel was healthy. The enterolith retrieved measured 2.5 × 1.5 cm (Fig. 5). Post-operative period was uneventful.

**Fig. 6:**
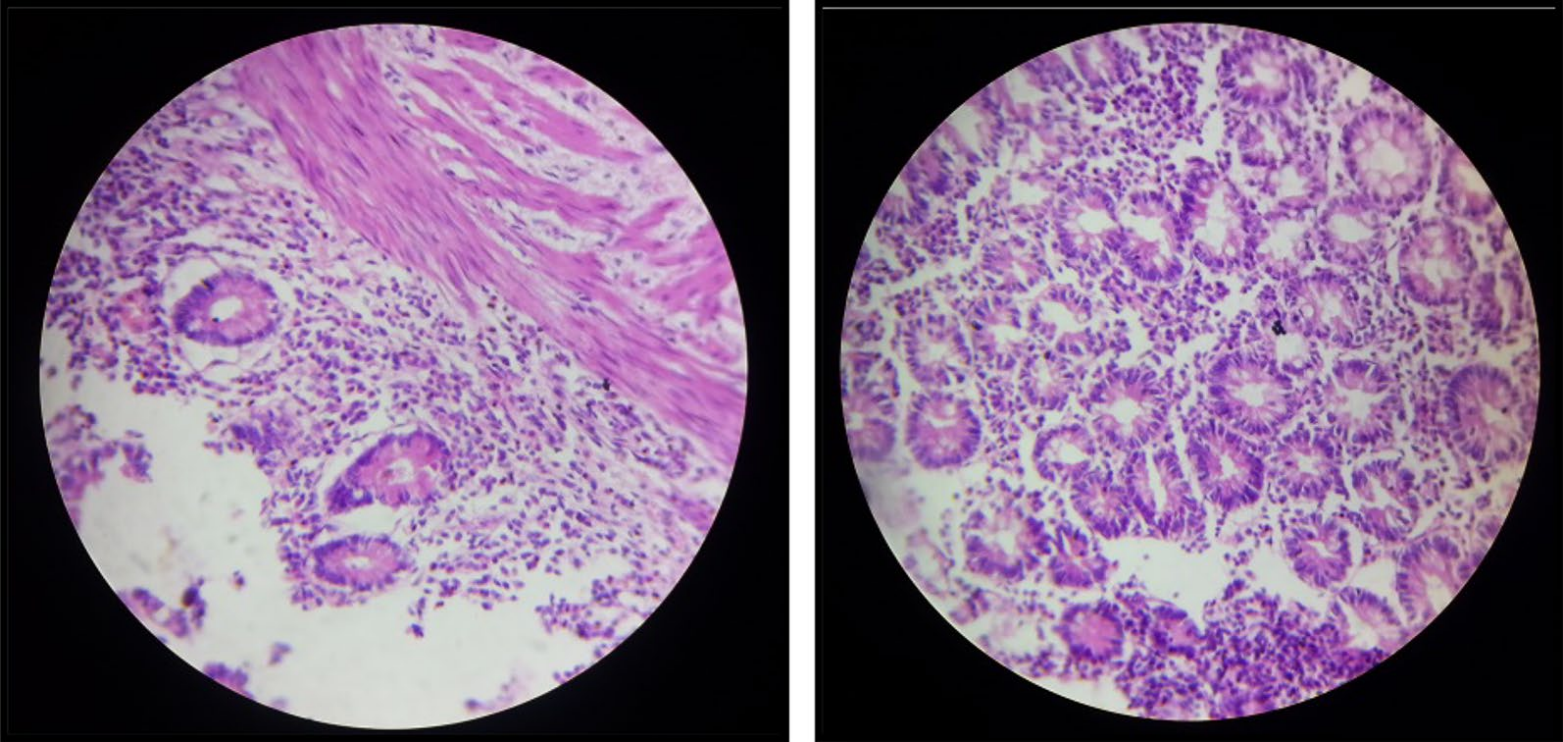
Histopathology showing eosinophilic and neutrophilic infiltrates in the ileal Mucosa and muscularis

The histopathology of the ileal specimen showed inflammatory cell infiltrate mainly neutrophils and eosinophils (Fig. 6). The eosinophilc infiltrates were mainly in the mucosal layer but also involved the muscularis. Intestinal Tuberculosis, Crohns disease, and malignancy were ruled out.

Patient is asymptomatic after one year of follow-up.

## Discussion

Primary enteroliths are formed in the small bowel and secondary enteroliths are formed in gallbladder as gallstones. (Yadav et al. 2015) True enteroliths of small intestine can be of 3 types depending on their composition—(1) those consisting of mainly bile acids, (2) those consisting of mainly phosphates, (3) those consisting of mainly calcium oxalate (D’souza et al. 2010; Shivathirthan et al. 2009). False endogenous enteroliths are common than true enterolith (Singleton 1970).

The site and pH of the intestinal lumen defines the chemical composition of enteroliths. The relatively high acidity of the proximal duodenum and jejunum allows precipitation of bile acids, particularly cholic acid, which are radiolucent. Precipitation of calcium in the lower distal parts of small intestine due to the alkaline pH in this region promotes formation of radio-opaque enteroliths (Oel-F et al. 2004).

Stasis, either due to stricture formation (intestinal tuberculosis, Crohn’s disease, carcinoid tumor, post-traumatic or post-surgical strictures, radiation enteritis, etc.) or diverticulae form the main reason for enterolith formation (Oel-F et al. 2004). Gamblin et al. (2003) and Jones and McWhirter (2010) reported cases of enterolith formation in Meckles diverticulum. Sureka et al. (2014) reported an interesting case of a radiopaque shadow in the pelvic region which was thought to be a vesical calculus but on further investigation was diagnosed as an enterolith in the ileal loop. In Crohns disease, multiple areas of small bowel stenosis are relatively common, but there are only few reported cases with stenosis complicated by enterolith. Geoghegan et al. (2005) reported a case of small bowel obstruction secondary to a giant enterolith in a patient of Crohn’s disease. Svanes and Halvorsen (1975) and Klinger et al. (1999) reported cases of enteroliths in jejunal diverticulae.

Most enteroliths are in apparent and cause no complications but sometimes may present with complications like intestinal obstruction, ileus or perforation. Klinger et al. (1999) in their article suggested that first therapeutic approach should be nonsurgical and surgery should be considered only if obstruction persists. Surgical management commonly involves enterotomy or occasionally resection.

## Conclusion

Strictures can be a cause of stasis causing primary enteroliths and should be considered while evaluating a case of Subacute intestinal obstruction.
